# Digital transformation of public health systems: strengthening to take rights seriously: Anabelle Macedo Silva

**DOI:** 10.1093/eurpub/ckac131.035

**Published:** 2022-10-25

**Authors:** A Macedo Silva, M Ventura

**Affiliations:** Public Health Institute, Federal University of Rio de Janeiro UFRJ, Rio de Janeiro, Brazil

## Abstract

The expansion of digital public health, with the use of data in digital systems for planning and operation of public health policy, presents itself as strategic for the public and digital future of Public Health Systems, such as Brazilian Sistema Único de Saúde/SUS, as well as for the effectiveness of the right to healthcare and expansion of the access to public health services (with the experience of COVID-19 highlighting the importance of digital health, as well as fostering its accelerated expansion). Such digital expansion will increasingly stress the fundamental right to the protection of personal data, and it is therefore important to strengthen the regulatory and care response of SUS in the field of digital public health, both for the preservation of its regulatory capacity in digital public health (facing market private interests of Big Tech for example), but also to guarantee the protection of fundamental rights, such as the protection of personal data. The theory of fundamental rights and the dogmatics on personal data protection offer support to solve the tensions to the right to data protection arising from the expansion of digital health, with consideration between the rights of patients-data subjects and the needs of managing and planning public health policy

**Key messages:**

• The intense use of data and digital systems in public health policy is strategic to strengthen public health systems.

• The enhancement of Public Health Systems’ regulatory capacity in digital transformation is important to protect right to health and data protection.

